# Zika virus non-structural protein NS2A mediated endoplasmic reticulum stress through interacting with Sarco/endoplasmic reticulum Ca^2+^-ATPase 2

**DOI:** 10.1128/jvi.00405-25

**Published:** 2025-06-23

**Authors:** Shan Wang, Shanshan Tang, Yuxin Zhou, Qifei An, Mengxing Du, Xiujuan Yang, Peng Zou, Li Tang, Yufeng Yu

**Affiliations:** 1 Shanxi Key Laboratory of Functional Proteins, School of Basic Medical Sciences and Pharmacy, Shanxi Medical University 74648, Taiyuan, China; 2 Department of Public Health Laboratory Sciences, School of Public Health, Shanxi Medical University 572534, Taiyuan, China; 3 Shanghai Public Health Clinical Center, Fudan University 12478, Shanghai, China; The Ohio State University, Columbus, Ohio, USA

**Keywords:** Zika virus, NS2A, SERCA2, ATP2A2, ER stress, Ca^2+ ^homeostasis

## Abstract

Zika virus (ZIKV) infection of neuronal cells leads to endoplasmic reticulum (ER) stress, which is one of the key causes of neuronal damage. However, how ZIKV mediates ER stress has not been fully understood. Here, we observed that ZIKV infection of astrocytes elevated Sarco/endoplasmic reticulum Ca^2+^-ATPase (SERCA) expression, increased intracellular Ca^2+^ concentration, and upregulated ER stress-related genes. SERCA2 was identified to regulate Ca^2+^ homeostasis and ER stress during ZIKV infection through both knockdown and overexpression of SERCA2 in astrocytes. Furthermore, ZIKV NS2A interacted with SERCA2 and increased the expression of SERCA2, disrupted Ca^2+^ homeostasis, and induced ER stress in astrocytes. After the knockdown of SERCA2 expression, Ca^2+^ homeostasis and ER stress were significantly mitigated in astrocytes expressing NS2A. Additionally, pTMS1-2 and pTMS4-5 of NS2A interacted with SERCA2 and regulated Ca^2+^ homeostasis and ER stress. ZIKV infection of the brains of BALB/c neonatal mice also elevated expression of SERCA2 and ER stress-related genes. Furthermore, SERCA2 expression facilitated ZIKV replication. These results suggested that ZIKV NS2A mediates ER stress through its interaction with SERCA2, providing new insights into the pathogenic mechanism of ZIKV and the development of anti-ZIKV therapies.

**IMPORTANCE:**

Zika virus (ZIKV) infection induces intracellular Ca^2+^ imbalance and endoplasmic reticulum (ER) stress. However, the molecular mechanisms involved in it remain unknown. Here we reported, for the first time, that ZIKV infection increased the expression of Sarco/endoplasmic reticulum Ca^2+^-ATPase 2 (SERCA2), which plays a crucial role in regulating Ca^2+^ homeostasis and ER stress. Furthermore, ZIKV NS2A was found to interact with SERCA2, contributing to the regulation of Ca^2+^ homeostasis and ER stress during ZIKV infection. And ZIKV NS2A pTMS1-pTMS2 and pTMS4-pTMS5 were the specific sites that interacted with SERCA2. These findings elucidate that the interaction between NS2A and SERCA2 is responsible for the regulation of the upstream signaling pathway of ER stress mediated by ZIKV infection. Additionally, the expression of SERCA2 promoted ZIKV proliferation, indicating that SERCA2 may serve as a potential target for anti-ZIKV therapies.

## INTRODUCTION

Zika virus (ZIKV) infection in pregnant women leads to microcephaly or abnormal neuronal development in infants, while in adults, it is associated with neurological disorders, such as Guillain-Barré syndrome, neuritis, or meningitis (1, 2). ZIKV infects neuronal cells and induces endoplasmic reticulum (ER) stress (3), triggering neurogenic arrest and unfolded protein response (UPR) dependent neuronal apoptosis, which contributes to the development of microcephaly (4, 5). ER stress refers to impaired proper protein folding and disrupted post-translational modifications in the endoplasmic reticulum, resulting in the accumulation of unfolded or misfolded proteins in the ER lumen (6, 7). UPR initiates programmed cell death when cells are under sustained ER stress (8). Inhibitors of the ER receptor PKR-like endoplasmic reticulum kinase (PERK) and inositol-requiring enzyme 1α (IRE-1α) effectively suppress the development of microcephaly caused by ZIKV infection (5). However, the mechanism by which ZIKV infection induces ER stress remains unclear.

The genome of ZIKV sequentially encodes three structural proteins (capsid protein, C; membrane protein and its precursor membrane, M/prM; envelope protein, E) and seven non-structural proteins (NS1, NS2A, NS2B, NS3, NS4A, NS4B, NS5) (9, 10). ZIKV NS2A consists of 226 amino acids with seven transmembrane segments (pTMS). The N-terminal region (pTMS1-pTMS2) is located near the ER lumen, while pTMS3 spans the ER membrane, and the C-terminal region (pTMS4-pTMS7) is close to the cytoplasm (11). NS2A plays a critical role in ZIKV replication, assembly, budding, and immune evasion (12). ZIKV NS2A disrupts neurogenesis in the cerebral cortex by degrading connexins and calcium adhesion proteins, leading to microcephaly (13). It should be noted that NS2A is highly expressed in the ER during ZIKV infection. Whether ZIKV NS2A plays a role in ZIKV-induced ER stress requires further investigation.

Sarco/endoplasmic reticulum Ca^2+^-ATPase (SERCA) is responsible for pumping cytoplasmic Ca^2+^ into the ER lumen, thereby maintaining a low concentration of cytoplasmic Ca^2+^ and a high concentration of Ca^2+^ in the ER lumen, which is essential for proper protein folding (14). Dysfunction of SERCA results in an imbalance of Ca^2+^ homeostasis, leading to the activation of Ca^2+^-sensitive proteases that degrade cellular components, ultimately causing necrosis or programmed cell death (15). Concurrently, depletion of Ca^2+^ in the ER lumen triggers ER stress (16–18). ZIKV infection elevates intracellular Ca^2+^ levels and induces neuronal cell death (19). SERCA may be involved in it. Ojha et al. have demonstrated that the SERCA-dependent ER stress pathway could serve as a target for inhibiting ZIKV replication (20). However, it is unknown whether ZIKV infection regulates SERCA.

Here, we observed that ZIKV infection elevated SERCA2 expression, which was accompanied by an imbalance in Ca^2+^ homeostasis and ER stress. The role of SERCA2 in regulating Ca^2+^ homeostasis and ER stress during ZIKV infection was demonstrated through both knockdown and overexpression of SERCA2. Furthermore, the pTMS1-2 and pTMS4-5 of NS2A mediated its interaction with SERCA2 and subsequent disruption of Ca^2+^ homeostasis and ER stress. Additionally, SERCA2 expression facilitated ZIKV replication. In conclusion, ZIKV NS2A mediated ER stress by hijacking SERCA2 to dysregulate Ca^2+^ homeostasis. This study provides new insights into the pathogenic mechanism of ZIKV and the development of antiviral therapies.

## RESULTS

### ZIKV infection increased SERCA2 expression, disrupted Ca^2+^ homeostasis, and triggered ER stress

**Fig 1:**
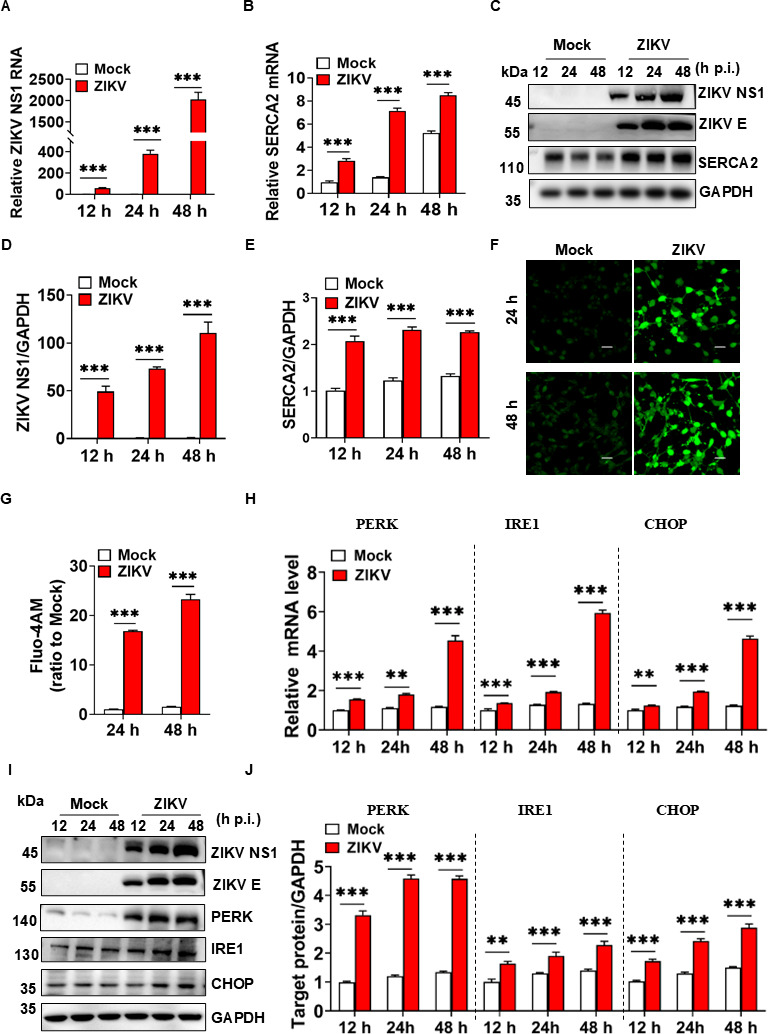
ZIKV infection increased SERCA2 expression, disrupted Ca^2+^ homeostasis, and induced ER stress. (**A–B**) U251 cells were infected with 0.1 MOI ZIKV/SZ01, and the cell samples were collected at 12, 24, and 48 h post-infection. qRT-PCR analysis showed viral NS1 gene and host SERCA2 gene expression. (**C–E**) The protein expression levels of SERCA2, ZIKV E, and ZIKV NS1 were detected by WB. The gray scale values of the protein expression bands in panel C were analyzed by ImageJ software (**D–E**). (**F–G**) U251 cells were infected with 0.1 MOI ZIKV/SZ01, and the cells were treated with Fluo-4 AM at 24 and 48 h post-infection. The changes in intracellular Ca^2+^ concentration were observed under the laser confocal microscope (**F**) and analyzed with ImageJ software (**G**). The scale bar represented 20 µm. (**H–J**) U251 cells were infected with 0.1 MOI ZIKV/SZ01, and the cell samples were collected at 12, 24, and 48 h post-infection. The expression levels of ER stress-related proteins, including PERK, IRE1, and CHOP, were detected using qRT-PCR (**H**) and western blot (**I**). The gray scale values of the protein expression bands in panel H were analyzed by ImageJ software (**J**). Cells in the group of mocks were not infected by ZIKV/SZ01 and used as the negative control. Data were statistically analyzed by one-way analysis of variance (ANOVA) and Sidak’s multiple comparisons test. Data are means ± SEM of triplicate experiments; **, *P* < 0.01; ***, *P* < 0.001.

SERCA2 is an endoplasmic reticulum Ca^2+^-ATPase (16). Dysregulation of SERCA2 results in the disruption of Ca^2+^ homeostasis and ER stress (21). To investigate whether ZIKV infection regulates the expression and function of SERCA2, U251 cells were infected with 0.1 multiplicity of infection (MOI) ZIKV/SZ01. The results showed that the gene and protein expression levels of SERCA2 were significantly higher in ZIKV-infected U251 cells than those of the mock group at 12, 24, and 48 h post-infection (Fig. 1A through E). The concentration of intracellular Ca^2+^ was significantly higher than that of the mock group, as detected by the Fluo-4 AM reagent (Fig. 1F and G). These data confirm that ZIKV infection causes an imbalance in intracytoplasmic Ca^2+^ homeostasis in U251 cells. The expression levels of ER stress-related proteins, including PERK, IRE1, and CHOP, were elevated after ZIKV/SZ01 infection (Fig. 1H through J), especially at 48 h. In conclusion, ZIKV infection promoted the expression of SERCA2, Ca^2+^ accumulation in the cytoplasm, and ER stress.

### SERCA2 regulated the imbalance of Ca^2+^ homeostasis and ER stress caused by ZIKV infection

**Fig 2:**
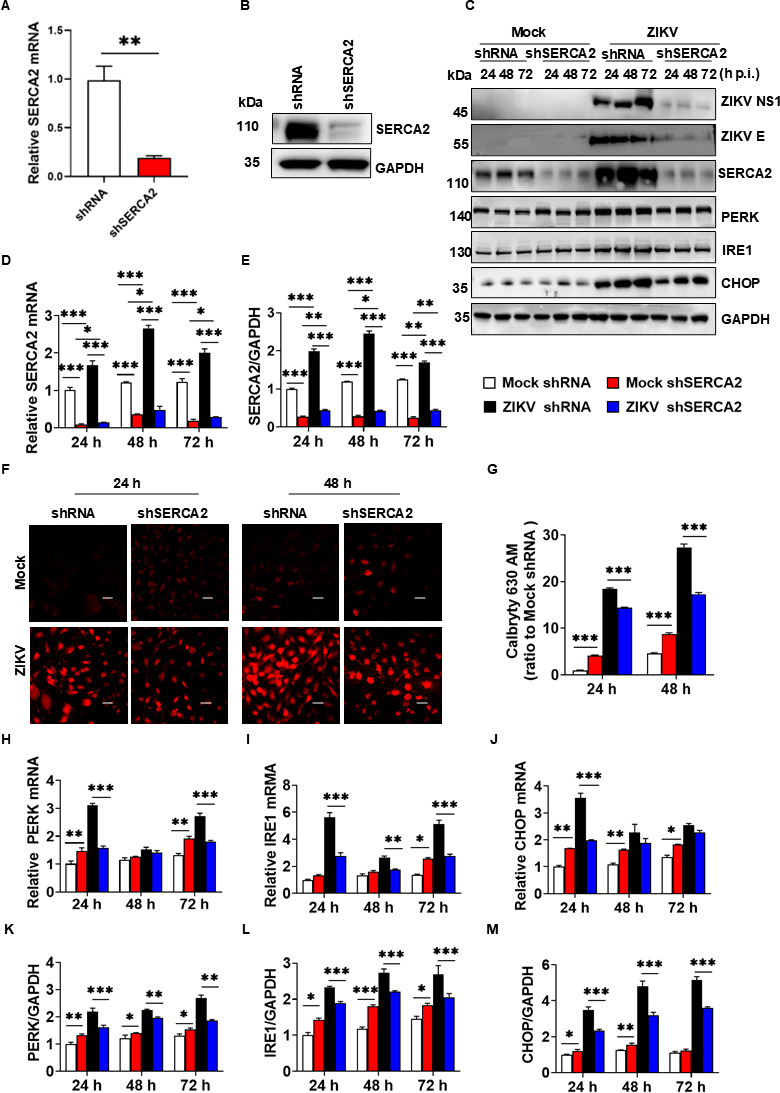
Knockdown of SERCA2 inhibited the imbalance of Ca^2+^ homeostasis and ER stress induced by ZIKV infection. (**A–B**) U251 stably knockdown of SERCA2 (shSERCA2) and the control (shRNA) cell lines were constructed by shRNA technology. The knockdown effect of SERCA2 was detected by qRT-PCR (**A**) and western blot (**B**). Data are means ± SEM of triplicate experiments, statistically analyzed by Student’s *t*-test, **, *P* < 0.01. (**F–G**) U251 shSERCA2 and shRNA cells were infected with 0.1 MOI ZIKV/SZ01, respectively. The cells were treated with Calbryte 630 AM at 24 and 48 h post-infection. The changes in intracellular Ca^2+^ concentration were observed under the laser confocal microscope (**F**) and analyzed by ImageJ software (**G**). The scale bar represented 20 µm. (**C–E, H–M**) U251 shSERCA2 and shRNA cells were infected with 0.1 MOI ZIKV/SZ01, and the cell samples were collected at 24, 48, and 72 h post-infection. The expression levels of SERCA2, ZIKV E, ZIKV NS1, and ER stress-related proteins, including PERK, IRE1, and CHOP, were detected using qRT-PCR (**D, H–J**) and western blot (**C**). The gray scale values of the protein expression bands in panel C were analyzed by ImageJ software (**E, K–M**). Cells in the group of mocks were not infected by ZIKV/SZ01. Data in panels D through M were statistically analyzed by one-way ANOVA and Tukey’s multiple comparisons test. Data are means ± SEM of triplicate experiments; *, *P* < 0.05; **, *P* < 0.01; ***, *P* < 0.001.

To investigate whether SERCA2 mediates the imbalance of Ca^2+^ homeostasis and ER stress, we constructed a U251 cell line of SERCA2 knockdown (shSERCA2 U251) and a control cell line (shRNA U251). As shown in Fig. 2A and B, the gene and protein expression levels of SERCA2 were significantly reduced in shSERCA2 U251 compared to those in the shRNA U251. Subsequently, we infected these cells with 0.1 MOI ZIKV/SZ01, and the concentration of intracellular Ca^2+^ was measured using Calbryte 630 AM. As shown in Fig. 2F and G, the concentration of intracellular Ca^2+^ in uninfected shSERCA2 U251 cells was higher than that in uninfected shRNA U251 cells. In contrast, when U251 cells were infected by ZIKV/SZ01, the concentration of intracellular Ca^2+^ in the shSERCA2 group was significantly lower than that of the shRNA group (Fig. 2F and G), confirming that SERCA2 was involved in the regulation of the imbalance of Ca^2+^ homeostasis caused by ZIKV infection. Additionally, we examined the expression levels of SERCA2 and ER stress markers in shSERCA2 U251 cells by western blot (WB) and quantitative real-time PCR (qRT-PCR). Figure 2D and E showed that ZIKV infection significantly promoted SERCA2 expression in both shSERCA2 and shRNA cells. Notably, during viral infection, the expression level of SERCA2 in shSERCA2 cells remains low (Fig. 2D and E). We also observed that the expression levels of the genes PERK (Fig. 2C, H and K), IRE1 (Fig. 2C, I and L), and CHOP (Fig. 2C, J and M) were slightly higher in uninfected shSERCA2 cells than those in uninfected shRNA cells. This indicated that knocking down SERCA2 could lead to an imbalance of intracellular Ca^2+^, triggering chronic ER stress protective mechanisms. However, the gene and protein expression levels of PERK, IRE1, and CHOP in the shSERCA2 group were lower than those in the shRNA group, especially at 24 h (Fig. 2C, H through M), suggesting that knockdown of SERCA2 could effectively inhibit ER stress induced by ZIKV infection.

**Fig 3:**
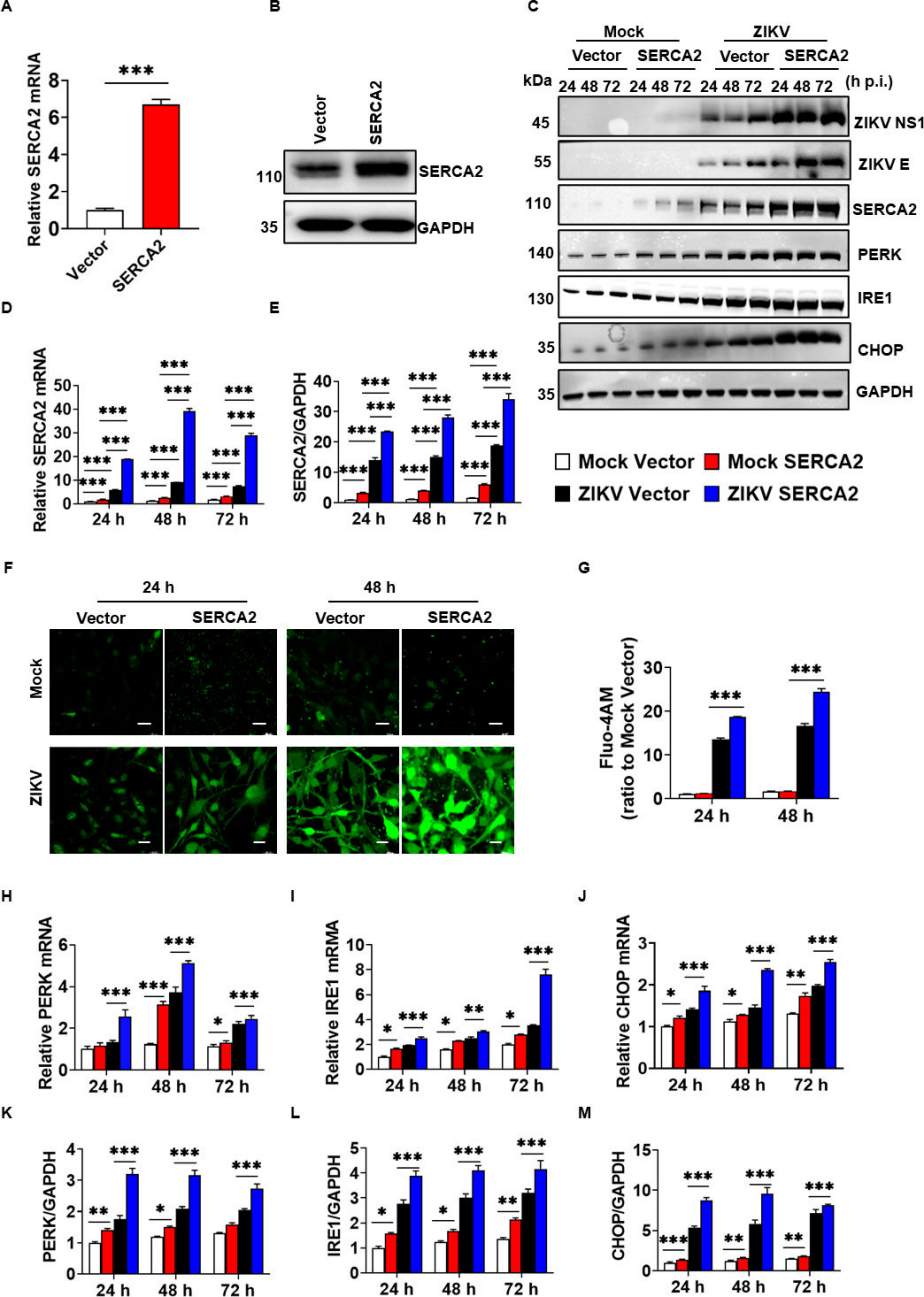
Overexpression of SERCA2 enhanced the imbalance of Ca^2+^ homeostasis and ER stress caused by ZIKV infection. (**A–B**) U251 stably overexpressed SERCA2 (SERCA2) and the control (Vector) cell lines were constructed by CRISPR/dCas9. The overexpression effect of SERCA2 was detected by qRT-PCR (**A**) and western blot (**B**). Data were statistically analyzed by Student’s *t*-test. Data are means ± SEM of triplicate experiments. ***, *P* < 0.001. (**F–G**) U251 SERCA2 and Vector cells were infected with 0.1 MOI ZIKV/SZ01, respectively. The cells were treated with Fluo-4 AM at 24 h and 48 h post-infection. The changes in intracellular Ca^2+^ concentration were observed under the laser confocal microscope (**F**) and analyzed by ImageJ software (**G**). The scale bar represented 20 µm. (**C–E, H–M**) U251 SERCA2 and Vector cells were infected with 0.1 MOI ZIKV/SZ01, and the cell samples were collected at 24, 48, and 72 h post-infection. The expression levels of SERCA2, ZIKV E, ZIKV NS1, and ER stress-related proteins, including PERK, IRE1, and CHOP, were detected using qRT-PCR (**D, H–J**) and western blot (**C**). The gray scale values of the protein expression bands in panel C were analyzed by ImageJ software (**E, K–M**). Cells in the group of mocks were not infected by ZIKV/SZ01. Data were statistically analyzed by one-way ANOVA and Tukey’s multiple comparisons test. Data are means ± SEM of triplicate experiments; *, *P* < 0.05; **, *P* < 0.01; ***, *P* < 0.001.

To further elucidate the role of SERCA2 in ZIKV infection, we employed CRISPR/dCas9 technology to construct stable cell lines expressing SERCA2 alongside control cells (Vector). The results demonstrated a significant increase in both mRNA and protein expression of SERCA2 (Fig. 3A and B), confirming the successful construction of the cell line. These engineered cells were subsequently infected by ZIKV/SZ01, and intracellular Ca^2+^ was monitored using Fluo-4 AM at 24 and 48 h post-infection. As illustrated in Fig. 3F and G, ZIKV infection led to a significant elevation in intracellular Ca^2+^ concentration compared to uninfected cells. Notably, SERCA2 overexpression exacerbated ZIKV-induced Ca²^+^ dyshomeostasis, showing a 24.45-fold increase in Ca²^+^ levels at 48 h compared to infected controls (Fig. 3F and G). We next analyzed SERCA2 and ER stress markers in ZIKV-infected SERCA2-overexpressing cells. SERCA2 expression was further upregulated in ZIKV-infected cells (Fig. 3C through E), alongside increased PERK, IRE1α, and CHOP levels (Fig. 3C, H through M). These data demonstrated that SERCA2 overexpression amplified ZIKV-driven Ca²^+^ dysregulation and ER stress. Collectively, these findings suggested that SERCA2 regulated the imbalance of Ca^2+^ homeostasis and ER stress caused by ZIKV infection.

### ZIKV NS2A interacted with SERCA2

**Fig 4:**
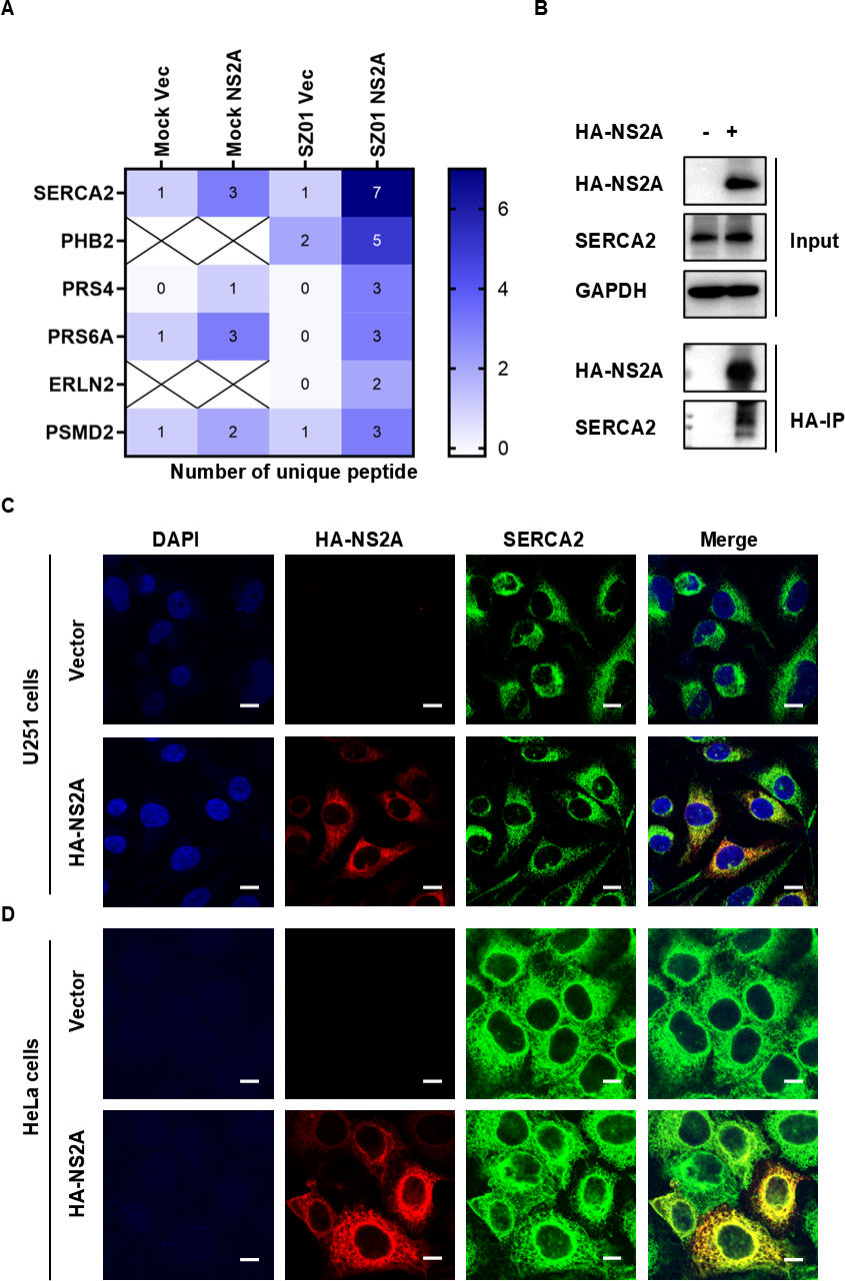
ZIKV NS2A interacted with SERCA2. (**A**) The U251 cell lines stably expressing HA-tagged ZIKV NS2A (U251 NS2A) and empty vectors (U251 Vector) were constructed by lentivirus. The above cells were infected with 0.1 MOI ZIKV/SZ01. After 24 h, the cell lysates were collected and analyzed according to the instructions of the Pierce Magnetic HA-Tag IP/Co-IP Kit. Subsequently, mass spectrometry was used to identify host factors that interacted with ZIKV NS2A. The protein was identified to interact with NS2A when the number of its unique peptides equaled or exceeded 2. (**B**) The cell lysates of U251 NS2A and U251 Vector cells without ZIKV were analyzed according to the instructions of the Pierce Magnetic HA-Tag IP/Co-IP Kit and western blot. (**C**) The co-localization of ZIKV NS2A (red) and SERCA2 (green) in U251 NS2A cells was observed using laser confocal microscopy. (**D**) HeLa cells were transfected with vectors expressing HA-NS2A of ZIKV and an empty vector. After 48 h, co-localization of HA-NS2A (red) and SERCA2 (green) was observed via laser confocal microscopy. The scale bar represented 10 µm. A group of empty vectors was set as a negative control.

Our previous study reported that ZIKV NS2A regulates ZIKV replication and that host factors may be involved (22). To analyze which host factors interact with ZIKV NS2A, U251 cells stably expressing NS2A (U251-NS2A) were infected with ZIKV/SZ01, and U251 cells expressing an empty vector (U251-Vec) were used as a negative control. Forty-eight hours later, the cell lysates were analyzed by co-immunoprecipitation (Co-IP) and mass spectrometry. The results indicated that SERCA2, PHB2, PRS4, PRS6A, ERLN2, and PSMD2 interacted with NS2A (Fig. 4A). In particular, three or seven unique peptides of SERCA2 were detected in U251-NS2A cells uninfected or infected by ZIKV/SZ01, respectively. However, only one unique peptide was found in U251-Vec cells (Fig. 4A). To confirm the interaction between NS2A and SERCA2, we conducted Co-IP and an immunofluorescence assay on U251-NS2A and U251-Vec cells. As illustrated in Fig. 4B and C, NS2A interacted and co-localized with SERCA2 in U251 cells. Additionally, NS2A was overexpressed in HeLa cells and was detected to be co-localized with SERCA2 (Fig. 4D). In conclusion, ZIKV NS2A interacted with SERCA2.

### ZIKV NS2A increased SERCA2 expression, disrupted Ca²^+^ homeostasis, and triggered ER stress

**Fig 5:**
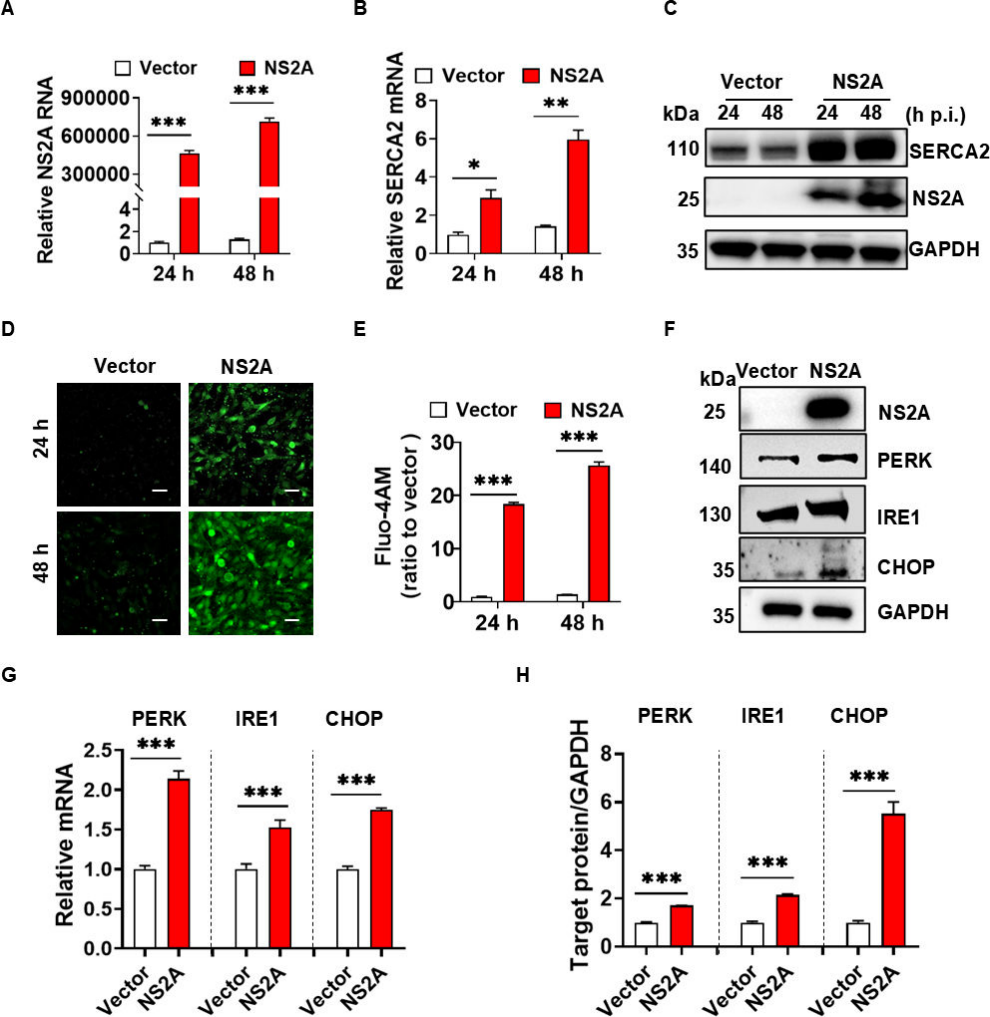
ZIKV NS2A increased the SERCA2 expression, disrupted Ca²^+^ homeostasis, and triggered ER Stress. (**A–C**) The U251 cell lines stably expressing HA-tagged ZIKV NS2A (U251 NS2A) and empty vectors (U251 Vector) were constructed by lentivirus. The above cells were treated with DMEM with 2% FBS for 24 and 48 h. The expression of NS2A and SERCA2 was detected by qRT-PCR (**A–B**) and western blot (**C**). (**D–E**) U251 NS2A and Vector cells were treated with DMEM with 2% FBS for 24 and 48 h, respectively. Fluo-4 AM was added to detect the changes in intracellular Ca^2+^ concentration. The cell samples were observed under the laser confocal microscope (**D**) and analyzed by ImageJ software (**E**). The scale bar represented 20 µm. Data are means ± SEM of triplicate experiments; *, *P* < 0.05; **, *P* < 0.01; ***, *P* < 0.001. (**F–H**) U251 NS2A and Vector cells were treated with DMEM with 2% FBS for 24 h. The expression levels of ZIKV NS2A and ER stress-related proteins, including PERK, IRE1, and CHOP, were detected using qRT-PCR (**G**) and western blot (**F**). The gray scale values of the protein expression bands in panel F were analyzed by ImageJ software (**H**). Data are means ± SEM of triplicate experiments; *, *P* < 0.05; **, *P* < 0.01; ***, *P* < 0.001.

ZIKV NS2A is an ER transmembrane protein that plays a critical role in ZIKV RNA replication and viral assembly (23). We demonstrated that ZIKV NS2A interacted with SERCA2 (Fig. 4). Does ZIKV NS2A contribute to the elevated expression of SERCA2, dysregulation of Ca^2+^ homeostasis, and ER stress during ZIKV infection? To address this question, we treated U251 cells stably expressing ZIKV NS2A (U251 NS2A) or empty vector (U251 Vector) with Dulbecco's modified Eagle medium (DMEM) containing 2% fetal bovine serum (FBS) for 24 or 48 h (Fig. 5A). As shown in Fig. 5B and C, the gene and protein expression levels of SERCA2 were significantly elevated in the U251 NS2A group compared to those in U251 Vector. Additionally, NS2A led to a marked increase in intracellular Ca^2+^ concentration, as shown in Fig. 5D and E. Furthermore, NS2A significantly elevated the mRNA and protein expression levels of PERK, IRE1, and CHOP, compared to the Vector group (Fig. 5F through H). In conclusion, ZIKV NS2A mediated a high expression of SERCA2, disrupted intracellular Ca^2+^ homeostasis, and initiated ER stress, which was consistent with ZIKV infection of U251 cells.

### SERCA2 regulated the imbalance of Ca^2+^ homeostasis and ER stress caused by ZIKV NS2A

**Fig 6:**
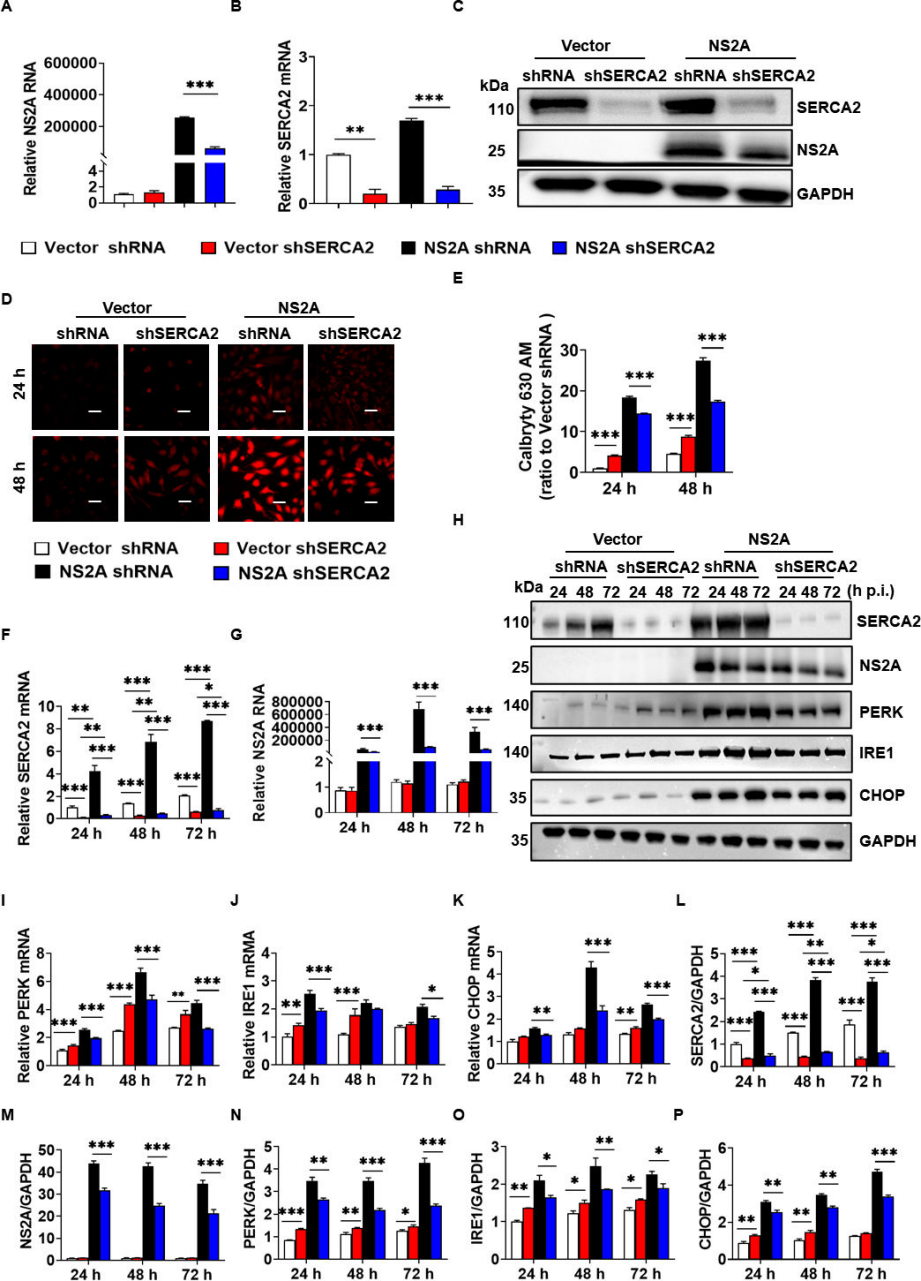
SERCA2 regulated the imbalance of Ca^2+^ homeostasis and ER stress caused by ZIKV NS2A. (**A–C**) Knockdown of SERCA2 in U251 cells stably expressing NS2A and Vector by shRNA technology, and corresponding shRNA control cells were constructed. Four cell lines were obtained, including U251 Vector shRNA, Vector shSERCA2, NS2A shRNA, and NS2A shSERCA2. The expression of NS2A and SERCA2 was detected by qRT-PCR (**A–B**) and western blot (**C**). (**D–E**) The above four cell lines were treated with DMEM with 2% FBS for 24 and 48 h, respectively. Calbryte 630 AM was added to detect the changes in intracellular Ca^2+^ concentration. The cell samples were observed under the laser confocal microscope (**D**) and analyzed by ImageJ software (**E**). The scale bar represented 20 µm. (**F–P**) The above four cell lines were treated with DMEM with 2% FBS for 24, 48, and 72 h. The expression levels of SERCA2, ZIKV NS2A, and ER stress-related proteins, including PERK, IRE1, and CHOP, were detected using qRT-PCR (**F–G, I–K**) and western blot (**H**). The gray scale values of the protein expression bands in panel H were analyzed by ImageJ software (**L–P**). Data were statistically analyzed by one-way ANOVA and Tukey’s multiple comparisons test. Data are means ± SEM of triplicate experiments. *, *P* < 0.05; **, *P* < 0.01; ***, *P* < 0.001.

To analyze whether ZIKV NS2A mediates the imbalance of Ca^2+^ homeostasis and ER stress through SERCA2, we performed SERCA2 knockdown in U251 NS2A or U251 Vector cells. Consequently, four U251 cell lines were constructed: Vector shRNA, Vector shSERCA2, NS2A shRNA, and NS2A shSERCA2 (Fig. 6A through C). Notably, SERCA2 knockdown suppressed the expression of NS2A, when compared to NS2A shRNA cells (Fig. 6B, C, G and M). Subsequently, we assessed the changes in intracellular Ca^2+^ concentration using Calbryte 630 AM. As depicted in Fig. 6D and E, knockdown of SERCA2 increased the concentration of Ca^2+^ in U251 Vector cells. However, the Ca^2+^ concentration in NS2A shSERCA2 cells was significantly reduced compared to NS2A shRNA cells. The effect of SERCA2 on NS2A-mediated ER stress was also investigated. As shown in Fig. 6F through P, SERCA2 knockdown increased the expression of PERK, IRE1, and CHOP in U251 Vector cells. Conversely, SERCA2 knockdown decreased the expression of PERK, IRE1, and CHOP in U251 NS2A cells, which was consistent with the effects observed during ZIKV infection (Fig. 2C, H through M). In conclusion, these results suggested that SERCA2 regulated the imbalance of Ca^2+^ homeostasis and ER stress induced by ZIKV NS2A.

### ZIKV NS2A pTMS1-pTMS2 and pTMS4-pTMS5 interacted with SERCA2

**Fig 7:**
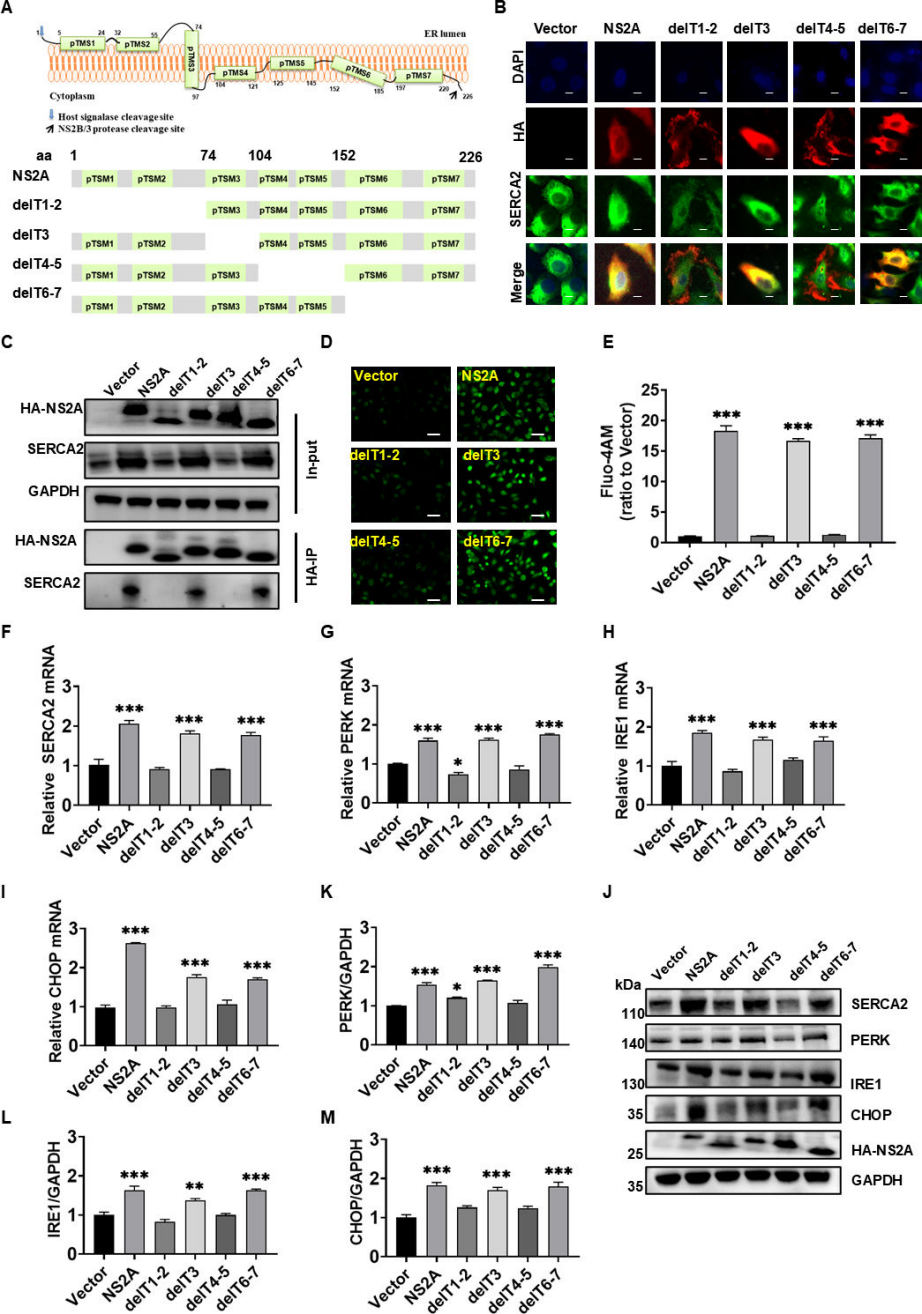
ZIKV NS2A pTMS1-pTMS2 and pTMS4-pTMS5 interacted with SERCA2. (**A**) Four truncators of ZIKV/SZ01 NS2A were constructed based on the transmembrane region of the ZIKV NS2A protein in the ER. NS2A sequence deleted 1-73 aa (delT1-2), 74-103 aa (delT3), 104-151 aa (delT4-5), 152-226 aa (delT6-7) were cloned into the vector of pRK5-HA, and the respective proteins were fused with an HA-tag at their carboxyl termini. (**B**) HeLa cells were transfected with vectors expressing NS2A truncators and NS2A. After 48 h, co-localization of truncators of NS2A (red) and SERCA2 (green) was observed via laser confocal microscopy. The scale bar represented 10 µm. (**C**) HEK293T cells were transfected with vectors expressing truncators of NS2A and NS2A. After 48 h, the cell lysates were analyzed according to the instructions of the Pierce Magnetic HA-Tag IP/Co-IP Kit and western blot using anti-SERCA2 mouse antibody or anti-HA rabbit antibody. (**D–E**) HeLa cells were transfected with vectors expressing truncators of NS2A and NS2A. After 48 h, Fluo-4 AM was added to detect the changes in intracellular Ca^2+^ concentration. The cell samples were observed under the laser confocal microscope (**D**) and analyzed by ImageJ software (**E**). The scale bar represented 20 µm. (**F–J**) HeLa cells were transfected with vectors expressing truncators of NS2A and NS2A. After 48 h, the expression levels of SERAC2 and ER stress-related proteins, including PERK, IRE1, and CHOP, were detected using qRT-PCR (**F–I**) and western blot (**J**). The gray scale values of the protein expression bands in panel J were analyzed by ImageJ software (**K–M**). Data were statistically analyzed by one-way ANOVA and Dunnett’s test. Data are means ± SEM of triplicate experiments; *, *P* < 0.05; **, *P* < 0.01; ***, *P* < 0.001. A group of empty vectors was set as a negative control.

To define the specific interaction between NS2A and SERCA2, we constructed four truncations based on NS2A pTMS (Fig. 7A). Immunofluorescence analysis revealed that delT1-2 and delT4-5 did not co-localize with SERCA2 (Fig. 7B). Furthermore, delT1-2 and delT4-5 failed to exhibit interaction with SERCA2 in the immunoprecipitation analysis (Fig. 7C). Subsequently, we analyzed the effects of the truncations of NS2A on SERCA2 function. The results demonstrated that deletion of pTMS1-pTMS2 and pTMS4-pTMS5 of NS2A failed to induce the imbalance in Ca^2+^ homeostasis (Fig. 7D and E). Additionally, following the deletion of pTMS1-pTMS2 and pTMS4-pTMS5, NS2A also failed to upregulate SERCA2 expression (Fig. 7F) and ER stress-related proteins (Fig. 7G through M). However, NS2A with deletion of pTMS3 and pTMS6-7 increased the expression of SERCA2, dysregulated Ca^2+^ homeostasis, and induced ER stress, which is consistent with the observed effects of NS2A. In conclusion, pTMS1-pTMS2 and pTMS4-pTMS5 of ZIKV NS2A mediated the interaction between NS2A and SERCA2.

### SERCA2 facilitated ZIKV replication

**Fig 8:**
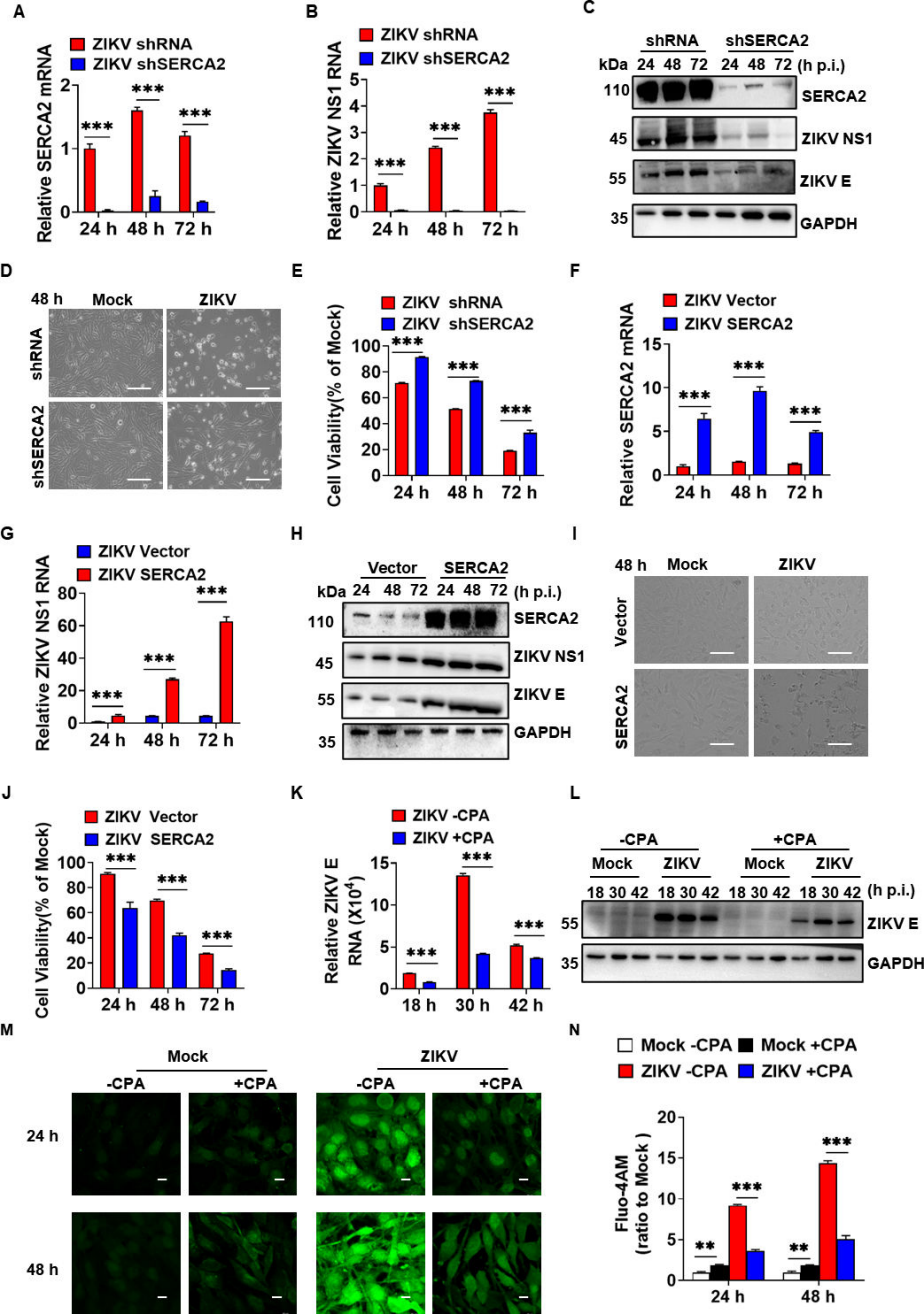
SERCA2 facilitated ZIKV replication. (**A–E**) U251 stably knockdown SERCA2 (shSERCA2) and its control (shRNA) cell lines were infected with 0.1 MOI ZIKV/SZ01, respectively. At 24, 48, and 72 h post-infection, the expression levels of SERCA2 and viral proteins (NS1 and E) were detected by qRT-PCR (**A–B**) and western blot (**C**). The cytopathic effect was observed under the light microscope (**D**), and the cell viability was assayed by CCK8. Data are means ± SEM (*n* = 6) (**E**). The scale bar represented 50 µm. (**F–J**) U251 stable overexpression of SERCA2 (SERCA2) and the control (Vector) cell lines were infected with 0.1 MOI ZIKV/SZ01, respectively. At 24, 48, and 72 h post-infection, the expression levels of SERCA2 and viral proteins (NS1 and E) were detected by qRT-PCR (**F–G**) and western blot (**H**). The cytopathic effect was observed under the light microscope (**I**), and the cell viability was assayed by CCK8, data are means ± SEM (*n* = 6) (**J**). The scale bar represented 50 µm. (**K–L**) With (+CPA) and without (−CPA) of cyclopiazonic acid (5 µM), U251 cells were infected with 0.1 MOI ZIKV/SZ01, respectively. At 18, 30, and 42 h post-infection, the expression level of ZIKV E was detected by qRT-PCR (**K**) and western blot (**L**). (**M–N**) With (+CPA) and without (−CPA) cyclopiazonic acid (5 µM), U251 cells were infected with 0.1 MOI ZIKV/SZ01, respectively. At 24 and 48 h post-infection, Fluo-4 AM was added to detect the changes in intracellular Ca^2+^ concentration. The scale bar represented 10 µm. The cell samples were observed under a laser confocal microscope (**M**) and analyzed by ImageJ software (**N**). Data were statistically analyzed by one-way ANOVA and Sidak’s multiple comparisons test. Data are means ± SEM of triplicate experiments. *, *P* < 0.05; **, *P* < 0.01; ***, *P* < 0.001.

Thapsigargin, a known SERCA2 inhibitor, has been reported to impede ZIKV infection (20). However, the direct impact of SERCA2 on ZIKV replication remains unknown. Here, U251 shSERCA2 or shRNA cells were infected by ZIKV/SZ01 (Fig. 8A). As shown in Fig. 8B and C, SERCA2 knockdown markedly inhibited the expression of ZIKV NS1 and E proteins. Moreover, cell viability significantly increased following the knockdown of SERCA2 during ZIKV infection (Fig. 8D and E).

Subsequently, we next overexpressed SERCA2 (Fig. 8F) to assess its impact on ZIKV replication and cell death. The results showed that the expression levels of ZIKV NS1 and E protein were significantly enhanced (Fig. 8G and H), and the cell viability was significantly reduced (Fig. 8I through J) in SERCA2 overexpression U251 cells. Lastly, we employed cyclopiazonic acid (CPA) (24) to inhibit SERCA2 activity, revealing that it diminished the expression of ZIKV E protein (Fig. 8K and L). Compared to the ZIKV infection without the CPA group, the addition of CPA effectively alleviated Ca²^+^ dyshomeostasis (Fig. 8M and N). Collectively, these results suggested that SERCA2 facilitated ZIKV replication and increased cell death induced by ZIKV infection.

### ZIKV infection of the brain of BALB/c neonatal mice elevated SERCA2 expression and ER stress

**Fig 9:**
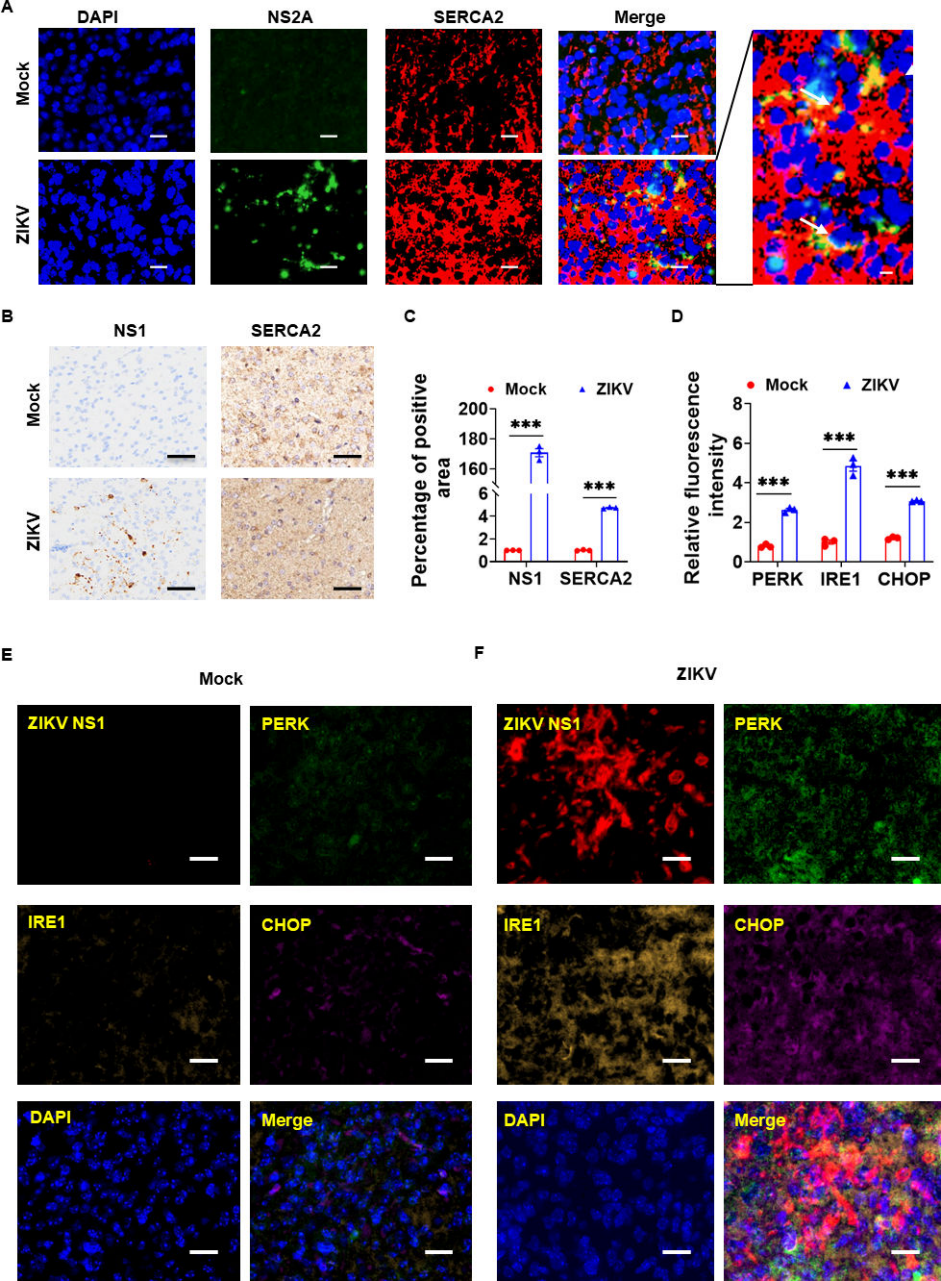
ZIKV infection of the brain of BALB/c neonatal mice elevated SERCA2 expression and ER stress. On day 1 after birth, BALB/c neonatal mice received an intracerebral injection of 20 µL of ZIKV/SZ01 at a concentration of 1 × 10^5^ p.f.u. or 0.9% NaCl (Mock). Seventy-two hours later, the brain tissues of neonatal mice were harvested for immunofluorescence or immunohistochemical analysis. (**A**) Co-localization of ZIKV NS2A (green) and SERCA2 (red) in the brain of BALB/c neonatal mice observed under laser confocal microscopy. The scale bar represented 20 µm, and the magnified merge icon scale was 10 µm. (**B–C**) The expression levels of ZIKV NS1 and SERCA2 were analyzed by immunohistochemistry (**B**), and the positive area was statistically analyzed using ImageJ software (**C**). The scale bar represented 100 µm. Data were statistically analyzed by one-way ANOVA and Sidak’s multiple comparisons test. Data are means ± SEM of triplicate experiments, ***, *P* < 0.001. (**D–F**) The expression levels of proteins associated with Zika virus (red) infection-induced ER stress-related proteins, including PERK (green), IRE1 (orange), and CHOP (pink), were detected using the tyramide signal amplification (TSA) method (**E–F**). The scale bar represented 20 µm. The average fluorescence intensity of the proteins was statistically analyzed by ImageJ software (**D**). Data were statistically analyzed by one-way ANOVA and Tukey’s multiple comparisons test. Data are means ± SEM of triplicate experiments, ***, *P* < 0.001.

To investigate the effects of ZIKV infection on SERCA2 expression and ER stress *in vivo*, we inoculated the brains of 1-day-old BALB/c neonatal mice with ZIKV/SZ01, referencing existing methods (25, 26). Three days post-infection, we collected the brains for immunofluorescence and immunohistochemical analysis. As illustrated in Fig. 9A, ZIKV NS2A co-localized with SERCA2 in the brains of the neonatal mice. Subsequently, we measured the expression of SERCA2 and ER stress-related proteins in the brains of BALB/c neonatal mice. The results showed that ZIKV infection increased the expression of SERCA2 (Fig. 9B and C) and significantly elevated the levels of ER stress-related proteins (Fig. 9D through F), including PERK, IRE1, and CHOP. These data demonstrated that ZIKV infection drove SERCA2 upregulation and ER stress in neonatal mouse brains.

## DISCUSSION

Here, we demonstrated that ZIKV infection upregulated SERCA2, which drove intracellular Ca²^+^ homeostasis dysregulation and triggered ER stress (Fig. 1). Genetic silencing of SERCA2 attenuated ER stress (Fig. 2), whereas overexpression of SERCA2 exacerbated ZIKV-induced Ca²^+^ imbalance and ER stress (Fig. 3), indicating that SERCA2 acts as a central regulator of ER stress signaling during ZIKV infection. We identified that pTMS1-pTMS2 and pTMS4-pTMS5 of ZIKV NS2A mediated direct interaction with SERCA2 (Fig. 4 to 7). Overexpression of SERCA2 enhanced viral replication and cell death (Fig. 8F through J), while SERCA2 knockdown suppressed ZIKV replication and reduced cell death (Fig. 8A through E). Inhibition of SERCA2 activity with CPA significantly attenuated ZIKV replication and restored Ca²^+^ homeostasis (Fig. 8K through N). These findings suggested that SERCA2 may represent a potential target for therapeutic intervention against ZIKV infection.

ZIKV is a mosquito-borne, strongly neurophilic flavivirus (27, 28), and infection can lead to disorders of neurogenesis, development, and maturation (29). However, ZIKV pathogenesis has not yet been clearly investigated. Replication of ZIKV induces ER remodeling and generation of viral replication compartments, which trigger the UPR, thereby tolerating infection or triggering apoptosis (30). ZIKV infection of brain-like organs accelerates Alzheimer’s disease via ER stress and UPR, including Aβ and p-Tau expression (31). When ZIKV infects a pregnant woman, it crosses the placental barrier and induces ER stress and death of placental trophoblast cells (32). Therefore, ZIKV-induced ER stress plays an important role in causing neurological disorders (33). However, how ZIKV induces ER stress lacks investigation. SERCA2, a major calcium transport protein, transports Ca²^+^ from the cytoplasm into the ER. SERCA2 dysfunction, when affected by physiological or pathological factors, disrupts intracellular Ca²^+^ homeostasis and induces ER stress, which mediates a variety of neurological disorders, such as bipolar disorder, schizophrenia, Parkinson’s disease, and Alzheimer’s disease (34, 35). Our study highlighted that ZIKV infection-induced ER stress was mediated by the interaction of ZIKV NS2A with SERCA2. We hypothesized that SERCA2 may also be involved in the neurological disorders caused by ZIKV infection, which warrants subsequent in-depth studies in the corresponding animal models.

ZIKV NS2A is a transmembrane protein localized to the ER, primarily involved in the formation of viral replication complexes (10). We observed that ZIKV NS2A interacted with SERCA2 (Fig. 4), enhancing SERCA2 expression and mediating Ca²^+^ homeostasis dysregulation and ER stress (Fig. 5 and 6), which aligned with ZIKV infection phenomena. Further investigations revealed that pTMS1-pTMS2 and pTMS4-pTMS5 of NS2A mediated the interaction between ZIKV and SERCA2 (Fig. 7). Notably, truncations lacking either NS2A pTMS1-pTMS2 (del1-2) or pTMS4-pTMS5 (del4-5) appeared to be localized to the cell membrane and exhibited distinct intracellular localization compared to NS2A del3 and del6-7, which localized to the ER (Fig. 7B). And NS2A del1-2 and del4-5 lost their ability to regulate SERCA2 expression, failing to induce significant Ca²^+^ homeostasis disruption and ER stress. Therefore, we hypothesize that the NS2A-SERCA2 interaction is closely associated with NS2A localization in the ER and may impair SERCA2 function, leading to compensatory upregulation of SERCA2 expression. Additionally, NS2A might regulate SERCA2 expression via binding to the SERCA2 promoter or enhancer regions. In conclusion, the molecular mechanisms underlying NS2A-mediated regulation of SERCA2 expression and function remain incompletely understood and need further study.

We also observed that intracellular Ca^2+^ and ER stress were sensitive to changes in the environment to which the cells were exposed. Intracellular Ca^2+^ levels and ER stress of cells (Mock or vector groups) were slightly increased with the prolongation of 2% FBS DMEM treatment (Fig. 1F through J), which may be related to nutrient and energy deficiencies. Astrocytes have a high energy demand, and their glucose oxidation rate is 4–10 times higher than the neuronal oxidation rate (36). SERCA2 transports Ca^2+^ from cytosol to endoplasmic reticulum, requires energy via ATP binding and hydrolysis (37). Energy deficiency inhibits SERCA2 function, leading to increased intracytoplasmic Ca^2+^ concentration and elevated levels of ER stress (38). We also observed that U251 cells in the mock group of Fig. 1I showed a gradual increase in ER stress as the culture proceeded, whereas U251 cells in the mock groups of Fig. 2, 3 and 6 showed relatively weak changes in ER stress. The different ER stress protein patterns may be due to the inherently different cell lines utilized in Fig. 1 and the other figures. Specifically, wide-type U251 cells were used in Fig. 1, while gene-edited U251 cells were employed in Fig. 2, 3 and 6. Gene editing may induce specific ER stress in U251 cells. As shown in Fig. 2 and 3, either knockdown or overexpression of SRECA2 slightly increased Ca^2+^ concentration and ER stress. And the ER stress induced by gene editing is more pronounced than that induced by DMEM with 2% FBS. Conversely, the ER stress resulting from ZIKV infection was notably significant, as evidenced by various detection methods. The use of DMEM with 2% FBS and gene editing did not influence the interpretation of the experimental results.

In conclusion, this study demonstrates that ZIKV NS2A-SERCA2 interaction disrupts Ca²^+^ homeostasis, thereby exacerbating ER stress and promoting ZIKV replication. Importantly, SERCA2 dysfunction emerges as a central driver of ZIKV pathogenesis, as its genetic or pharmacological inhibition significantly attenuated viral replication and restored Ca²^+^ homeostasis. These findings elucidate a novel mechanism by which ZIKV coopts host calcium regulatory machinery to fuel infection, positioning SERCA2 as a promising therapeutic target for antiviral intervention.

## MATERIALS AND METHODS

### Cells and viruses

The human astrocyte cell line (U251), Vero, and HEK293T cells were cultured in Dulbecco’s Modified Eagle’s Medium (DMEM, Gibco), supplemented with 10% fetal bovine serum (FBS, ExCell Bio), 100 U/mL of penicillin and streptomycin (Gibco) in an incubation chamber with 5% CO_2_ at 37°C. The ZIKV/SZ01/2016 strain (GenBank number: KU866423) was isolated from a patient returning from Samoa and amplified in Vero cells (39). The viral titers were determined by virus plaque assay (39).

### Construction of cell lines

U251 cells stably expressing the ZIKV NS2A (U251-NS2A) protein and control vector (U251-Vec) were constructed in our previous studies (22). To construct SERCA2 knockdown cell lines, pGIPZ-shSERCA2-puro plasmids and pGIPZ-puro empty plasmid were acquired from Shanghai Jiao Tong University. Three shRNA sequences targeting SERCA2 (NM_170665.4) were used: 5′-CTGGTGATATTGTAGAAAT-3′, 5′-AGAAAGTCAATGTCGGTTT-3′, and 5′-AGGTGATACTTGTTCCCTT-3′. Cells were maintained in DMEM with 5 µg/mL puromycin (MCE).

To knock down SERCA2 in U251 cells stably expressing NS2A, the puromycin resistance in pGIPZ-shSERCA2-puro and pGIPZ-puro plasmids was replaced with G418 resistance. Four cell lines were generated: Vector shSERCA2, Vector shRNA, NS2A shSERCA2, and NS2A shRNA. Cells were maintained in DMEM medium with 5 µg/mL puromycin (MCE) and 500 µg/mL G418 (Thermo).

To achieve SERCA2 overexpression in U251 cells, the CRISPR/dCas9 system was utilized (40), including LentiMPH v2-hygro and LentisAM v2-Blasticidin (Addgene). The sequences of sgRNAs for SERCA2 (Gene ID: 488) were 5′-CTGGTGATATTGTAGAAAT-3′, 5′-AGAAAGTCAATGTCGGTTT-3′, 5′-AGGTGATACTTGTTCCCTT-3′. The empty lentiSAM v2-Blasticidin vector served as a negative control for the overexpression of SERCA2 cells. Cells were maintained in DMEM medium with 250 µg/mL of hygromycin B (Thermo) and 1.5 µg/mL of Blasticidin (Thermo).

To verify the successful construction of the above cells, we conducted qRT-PCR and western blot analyses.

### Detection of [Ca^2+^] levels in the cell cytoplasm

To precisely monitor real-time changes in intracellular calcium concentration ([Ca²^+^]i) during viral infection, we employed Fluo-4 AM (41) and Calbryte 630 AM (42), two high-sensitivity fluorescent probes with distinct spectral profiles. Calbryte 630 AM was specifically selected to circumvent spectral overlap with GFP fluorescence in shSERCA2 knockdown cell lines. Cells seeded in laser confocal dishes were cultured in 5% CO_2_ at 37°C for 24 h, then infected with 0.1 MOI ZIKV or treated with 2% FBS DMEM. At 24 h or 48 h post-infection, we removed the supernatant, washed cells three times with HEPES buffer, and loaded them with Fluo-4 AM (Solarbio, 5 µM) or Calbryte 630 AM (AAT Bioquest, 5 µM) in HEPES for 30 min at 37°C (protected from light). To ensure complete de-esterification, cells were incubated with 1% FBS-HEPES for 40 min, followed by five HEPES washes to remove extracellular dye. Live-cell Ca²^+^ fluorescence was imaged on an Olympus FV3000 confocal microscope.

### Construction of NS2A truncators

ZIKV NS2A truncations were constructed based on the seven transmembrane regions of NS2A, as follows: delT1-2, NS2A deleted 1-73 aa; delT3, NS2A deleted 74-103 aa; delT4-5, NS2A deleted 104-151 aa; delT6-7, NS2A deleted 152-226 aa. All the above sequences were cloned into the pRK5-HA vector, expressed in fusion with the HA tag.

### Quantitative real-time polymerase chain reaction

To assess the gene expression of target proteins in the cells, total cellular RNA was extracted using TRIzol (Thermo) after the cells had been treated. The Prime Scripter Scientific kit (TaKaRa) was used to transcribe 1 µg of total RNA into cDNA. Real-time quantitative PCR reactions were performed using the TB Green Premix Ex Taq II kit in a 20 µL system on a Quant Studio 6 PCR instrument (Thermo). Data analysis was performed using the 2^−ΔΔCt^ method. The primers used here were listed in Table 1.

**TABLE 1:** Sequences of primers for qRT-PCR used in the study

Accession number	Name	Sequence
NM_001313915.2	H-PERK-F	5′-ATCGCAGAGGCAGTGGAGTT-3′
	H-PERK-R	5′-CATAAGCTGGCATTGGGGTC-3′
NM_001433.5	H-IRE1-F	5′-CCTCCGAGCCATGAGAAATAA-3′
	H-IRE1-R	5′-GGGGAAGCGAGATGTGAAGT-3′
NM_001195053.1	H-CHOP-F	5′-CAGCCACTCCCCATTATCCT-3′
	H-CHOP-R	5′-ACCTCTTGCAGGTCCTCATACC-3′
NM_170665.4	H-SERCA2-F	5′-TCCGCTACCTCATCTCGTCCAA-3′
	H-SERCA2-R	5′-CGTCAGCAGCAATGAACCACCA-3′
KU866423	ZIKV-NS1-F	5′-GGTGCTCGGTGGACTTCTCAA-3′
	ZIKV-NS1-R	5′-TGACTGCTGCTGCCAATCTACG-3′
KU866423	ZIKV-NS2A-F	5′-GGTTGGCAATACGAGCGATGGT-3′
	ZIKV-NS2A-R	5′-TCCTTGTGAGCAACAGCAGTCC-3′
KU866423	ZIKV-E-F	5′-GGGTTGATGTTGTCTTGGAACAT-3′
	ZIKV-E-R	5′-AGGCTTCACCTTGTGTTGGG-3′
NM_001101.5	H-β-actin-F	5′-AAGGAGAAGCTGTGCTACGTCGC-3′
	H-β-actin-R	5'AGACAGCACTGTGTTGGCGTACA-3′

### Western blot analysis

To test the expression of target proteins, cells were lysed with lysis buffer with protease inhibitors (Roche). The cell lysates were immunoblotted with the indicated antibodies. Anti-ZIKV E and anti-ZIKV NS1 antibodies were purchased from Gene Tex. Anti-HA and anti-PERK antibodies were purchased from CST. An anti-SERCA2 antibody was purchased from Abcam. Anti-IRE1, anti-CHOP, and anti-GAPDH antibodies were purchased from Proteintech.

### Co-immunoprecipitation assay

To verify the interaction of ZIKV NS2A with SERCA2, U251 stably expressing HA-NS2A and U251 with an empty vector were collected for co-immunoprecipitation according to the manufacturer’s instructions of Pierce anti-HA magnetic beads (Thermo). To identify the specific interaction region of ZIKV NS2A with SERCA2, HEK293T cells were seeded into 6-well plates and cultured at 37°C with 5% CO_2_ for 24 h. The cells were transfected with pRK5-HA-NS2A, delT1-2, delT3, delT4-5, delT6-7, or empty pRK5-HA by VigoFect transfection reagent (Vigorous). Forty-eight hours later, cell samples were collected for immunoprecipitation assay according to the manufacturer’s instructions of Pierce anti-HA magnetic beads (Thermo). The final samples were analyzed by WB.

### Mass spectrometry

To clarify the host factors that interact with NS2A, U251 cells expressing ZIKV NS2A or empty vector were treated with 0.1 MOI ZIKV/SZ01 in DMEM with 2% FBS or DMEM with 2% FBS. Samples were collected at 48 h post-infection. Immunoprecipitation (IP) experiments were conducted using Pierce anti-HA magnetic beads (Thermo), and 120 µL of sample buffer was collected. SDS-PAGE electrophoresis was performed using 80 µL of samples for each gel well, and the process was halted immediately after the samples penetrated 3 mm into the separation gel. The target proteins in the gel were collected for mass spectrometry assay by the public technology service platform of Shanghai Medical College at Fudan University.

### Immunofluorescence assay

U251 cells expressing ZIKV NS2A or empty vector were seeded onto coverslips in 24-well plates and incubated at 37°C with 5% CO_2_ for 24 h. Cells were fixed with 4% paraformaldehyde for 10 min, then perforated with 0.1% Triton X-100 for 15 min and blocked with 3% BSA for 30 min. Thereafter, 1% BSA diluted anti-HA Tag mouse antibody (CST) or anti-SERCA2 rabbit antibody (Abcam) was added to the cells and incubated for 1 h at room temperature. After three washes with PBS, Alexa Fluor 488-labeled donkey anti-rabbit IgG and Alexa Fluor 594-labeled donkey anti-mouse IgG (Thermo) were added to the cells and incubated at room temperature for 1 h. After five washes with PBS, the coverslips were sealed with DAPI (Thermo) and subsequently analyzed under an FV3000 Olympus laser confocal microscope.

HeLa cells were transfected with pRK5-HA-NS2A or empty pRK5-HA for 48 h. Immunofluorescence staining was performed using anti-HA Tag mouse antibody (CST) or anti-SERCA2 rabbit antibody (Abcam), and Alexa Fluor 488-labeled donkey anti-rabbit IgG and Alexa Fluor 594-labeled donkey anti-mouse IgG (Thermo).

HeLa cells were transfected with pRK5-HA-NS2A, delT1-2, delT3, delT4-5, delT6-7, or empty pRK5-HA for 48 h. Immunofluorescence staining was performed using anti-HA Tag rabbit antibody (CST), anti-SERCA2 mouse antibody (Abcam), and Alexa Fluor 594-labeled donkey anti-rabbit IgG and Alexa Fluor 488-labeled donkey anti-mouse IgG (Thermo).

### Inhibitory effects of CPA on ZIKV and Ca^2+^ homeostasis

To evaluate the inhibitory effect of CPA on ZIKV, U251 cells were infected with 0.1 MOI ZIKV/SZ01 with and without CPA (5 µM), respectively. Supernatants were collected at 18, 30, and 42 h post-infection, and viral RNA copy numbers were quantified using established experimental methods (22). Additionally, the expression of the viral envelope (E) protein was detected by WB using an anti-ZIKV E antibody (Gene Tex).

To investigate the effect of CPA on Ca^2+^ homeostasis, U251 cells were infected with 0.1 MOI ZIKV/SZ01 with and without CPA (5 µM), respectively. Changes in intracellular Ca^2+^ levels were monitored using Fluo-4 AM at 24 h and 48 h post-infection, and the fluorescence analysis of Ca^2+^ was conducted by ImageJ software.

### Animal experiments

Pregnant BALB/c mice (E16, *n* = 6) were randomly assigned to two groups. On the first day after birth, BALB/c neonatal mice received an intracerebral injection of 20 µL of ZIKV/SZ01 at a concentration of 1 × 10^5^ p.f.u. or 0.9% NaCl. Seventy-two hours later, the brain tissues of neonatal mice were collected for immunofluorescence to analyze the interactions between SERCA2 and ZIKV NS2A. Additionally, immunohistochemistry was performed to examine ZIKV infection and SERCA2 protein expression. Furthermore, to observe the expression of three ER stress genes, including PERK, IRE1, and CHOP, at the same location of viral infection, the tyramide signal amplification (TSA) method was utilized. TSA relies on HRP-catalyzed tyramide activation. In the presence of H₂O₂, HRP oxidizes fluorophore-conjugated tyramide into highly reactive radicals, which covalently bind to nearby proteins (e.g., tyrosine residues). Each HRP molecule generates hundreds of tyramide deposits, enabling localized signal amplification with high specificity and minimal background. This method enhances sensitivity and is widely used in IHC, FISH, and ultrasensitive detection (43, 44). Mouse anti-ZIKV NS1 antibody was utilized as the primary antibody, while anti-mouse IgG-HRP served as the secondary antibody. The samples were washed with TBST. Subsequently, fluorophore-labeled tyramide was added dropwise for 10 min in the absence of light and was then washed with TBST. Microwave-assisted elution was conducted to dissociate the ZIKV NS1 antibody-HRP complex. After cooling, anti-PERK, anti-IRE1, and anti-CHOP antibodies, along with fluorophore-labeled tyramide, were replaced, and the aforementioned steps were repeated, respectively. Finally, a DAPI working solution and an anti-quenching sealer were added dropwise, and images were captured using a multispectral imaging system, along with slide viewer software for overlay channel analysis. Here, Anti-PERK, anti-IRE1, and anti-CHOP antibodies were purchased from Abclonal. ZIKV NS2A antibody was prepared by our team previously (45). The other antibodies were derived from the same sources as those in WB.

### Statistical analysis

All data were analyzed using GraphPad Prism 9 software (La Jolla, CA, USA). When the data were normally distributed and with no significant difference in homogeneity of variance, Student’s *t*-test was employed to assess the difference between the two groups. For three or more groups, the data were analyzed by one-way analysis of variance (ANOVA). Then, the multiple comparisons were performed using Sidak’s multiple comparisons test, Tukey’s multiple comparisons test, or Dunnett’s test, according to GraphPad Prism 9 software. All experiments included at least three independent biological replicates. The results are presented as means ± SEM, with significance levels indicated as follows: *, *P* < 0.05; **, *P* < 0.01; ***, *P* < 0.001.

## Data Availability

The data sets analyzed during the current study are available from the corresponding author on reasonable request.
